# Food-Insecure Household’s Self-Reported Perceptions of Food Labels, Product Attributes and Consumption Behaviours

**DOI:** 10.3390/nu11040828

**Published:** 2019-04-12

**Authors:** Lucy M. Butcher, Maria M. Ryan, Therese A. O’Sullivan, Johnny Lo, Amanda Devine

**Affiliations:** 1 School of Medical and Health Sciences, Edith Cowan University, Joondalup, WA 6027, Australia; t.osullivan@ecu.edu.au (T.A.O.); a.devine@ecu.edu.au (A.D.); 2 Foodbank Western Australia, Perth Airport, WA 6105, Australia; 3 School of Business and Law, Edith Cowan University, Joondalup, WA 6027, Australia; m.ryan@ecu.edu.au; 4 School of Science, Edith Cowan University, Joondalup, WA 6027, Australia; j.lo@ecu.edu.au

**Keywords:** vulnerable groups, food poverty, food insecurity, food literacy, public health

## Abstract

Dietary compromises related to food insecurity profoundly undermine health and constitute a serious public health issue, even in developed nations. The aim of this study was to explore the impact of food labelling and product attributes on the purchasing choices of food-insecure households in Australia. An online survey containing 19 food choice and 28 purchasing behaviours questions was completed by 1056 adults responsible for household grocery shopping. The short form of the US Household Food Security Survey Module was used as the food security indicator. Multinomial logistic regression modelling was employed to analyse the survey data. Respondents were classified as having either high-marginal (63.4%, *n* = 670), low (19.8%, *n* = 209) or very low (16.8%, *n* = 177) food security. Respondents with low or very low food security status were less likely to self-report understanding the information on the back of packaging (*p* < 0.001), find information on food labels useful (*p* = 0.002) or be influenced by product nutrition information (*p* = 0.002). Convenience (*p* < 0.001), organic (*p* = 0.027) and supermarket-branded products (*p* < 0.001) were more likely to be rated as important by food-insecure respondents when compared to their food-secure counterparts. When asked to rate “how healthy” their diet was, high–marginal FS respondents were twice as likely describe their diet as healthy than very low FS respondents (*p* = 0.001).

## 1. Introduction

A nutritionally adequate diet is increasingly viewed as the leading modifiable factor in the prevention of chronic disease [1]. Regardless of this, the majority of Australians have suboptimal intakes of nutrient dense foods, such as fruit and vegetables, and compliance to the national dietary guidelines is low [2]. Food-insecure households are thought to be a particularly vulnerable subpopulation and are more likely exhibit a poorer diet quality than the general Australian population [3,4,5,6]. Food insecurity exists “whenever the availability of nutritionally adequate and safe foods, or the ability to acquire acceptable food in socially acceptable ways, is limited or uncertain” [7]. The current reported national prevalence of food insecurity is 3.7% [2]. However, other Australian researchers have found the rate of food insecurity to be as high as 25% [8] to 36% [9] when a more sensitive food insecurity measure has been applied.

Food insecurity occurs on a continuum of severity which may exist with or without hunger depending on the scarcity of nutritious food. Individuals experiencing mild or “low” food security may try to avoid hunger by reducing portion sizes, skipping meals, choosing cheaper foods or reducing diet variety [10]. Reduced intake of fruit and vegetables, overconsumption of high energy, nutrient-poor foods and decreased diet diversity, are frequently viewed as characterising features of mild forms of food insecurity in developed nations [11,12,13]. However, when food is simply not available, coping strategies prove ineffective and hunger is inevitable. Food insecurity with hunger is synonymous with severe food insecurity or “very low” food security (FS) [10]. The dietary compromises related to even mild forms of food insecurity have been cited as significant, profoundly undermining health [8,14]. When prevalence is considered, food insecurity constitutes a serious public health issue, even in developed nations such as Australia [15].

Recently, as a strategy to improve purchasing behaviours and address poor dietary intakes of Australians, there has been conscious effort to improve and simplify food labelling (particularly front of pack information including nutrition claims) [16]. In other developed nations, the majority of consumers report using food labels to assist in food choices [17,18]. While interest in food labels may be high, Sharf, et al. [19] suggests the public’s actual understanding of the topic is low.

The nutritionally inferior dietary patterns of food-insecure people are generally regarded as a symptom of economic constraints [11]. There is limited research about other factors (such as food labels and product attributes) influencing food choices and the flow-on effect to consumption behaviours in this subpopulation. To the authors’ knowledge, no Australian research has been conducted into food-insecure households’ self-reported use or understanding food labels. In addition, only one study outside Australia has investigated this, specifically how food-insecure people residing in America interpret food labels [20]. This research forms one component of a multidisciplinary study investigating food shopping and consumption behaviours. The purpose of this article is to explore the impact of food labelling and product attributes on the food choices of food-insecure individuals. 

## 2. Materials and Methods

The aims of this study were to: (1) Assess the extent to which food-insecure people find food labels useful when making food choices, (2) determine if other product attributes, such as cost and quality, affect purchasing choices and (3) consider how these factors may translate into consumption behaviours.

An online survey investigating food choices and purchasing behaviours was conducted between November 2014 and February 2015. All participants were recruited through a commercial research company, and survey administration was through Qualtrics (Provo, Utah, USA). Respondents were required to be over 18 years of age, the main household grocery purchaser and located in one of five Australian states (New South Wales, Victoria, Western Australia, South Australia and Queensland). As the survey was disseminated online, internet access was necessary for participation. No further inclusion or exclusion criteria were applied. In Australia, approximately a third of primary household grocery shoppers are thought to be male [21]. Therefore, an adequate quota for male representation was set at a minimum proportion of 30% in an effort to make the sample population comparable to the Australian general population. Quotas were also established for age and location, in order to align with the general Australian population [22]. 

### 2.1. Survey Content

The survey comprised: Twelve sociodemographic questions (including age, gender, immigration, occupation, education, household income, household structure and marital status), the six-item US Household Food Security Survey Module (HFSSM), twenty-eight food purchasing and nineteen consumption questions. 

The six-item HFSSM was included as a food security indicator. The short form was selected due to the comparable accuracy with the longer 18 item form (correctly identified 97.7% of households), while simultaneously having the added benefit of reduced respondent burden [23]. Food security (FS) status was categorised and named in accordance with the HFSSM user notes [24], and the authors’ previous research outlines a more detailed methodology [9]. The three levels of household FS referred to in this study are: High-marginal FS (no anxiety about accessing adequate food), low FS (reduced quality, variety and desirability of diet, but no reduction in quantity of food) and very low FS (intermittent disruption of eating patterns and reduced food intake of one or more household members) [10]. For the purpose of this research, food insecurity is considered a broader term encompassing households reporting both low and very low FS [24]. 

The food purchasing behaviour indicators consisted of a total of 28 questions: Eleven on product attributes, nine on food label reading and an additional eight on nutrition claims. The selection of product attributes questions was influenced by an extensive literature review, not included in this paper, of the associated factors of food insecurity. Food label reading questions were developed to demonstrate how useful consumers found nutrition information on packaging. Questions relate to both the front of pack nutrition claims and the back of pack information (including the nutrition information panel and the ingredient list). In Australia, nutrition claims are statements about the content of particular substances or nutrients in a food product, for example “low fat” or “high protein” [25]. Nutrition claims reflected eight commonly used terms on food packages at the time of survey administration: Low glycaemic index, kilojoules, sugar, preservatives, carbohydrates, saturated fats, sodium and high protein [26].

Food and beverage consumption questions were based on the standard serve or serving sizes outlined in the 2013 Australian Dietary Guidelines [27]. Respondents were asked to estimate the number of standard serves consumed for each food grouping on a typical day. Foods and drinks were classified into 18 distinct groupings for analysis. In conjunction with the dietary intake questions, respondents were also asked to rate the perceived healthiness of their diet. 

### 2.2. Statistics

Response categories for the food purchasing questions were collapsed from a five-point scale (strongly agree to strongly disagree) to a three-point scale (agree, neither agree nor disagree and disagree), due to low cell counts in some categories. The response categories for the 18 food consumption indicators are defined by a five-point scale (0, 1, 2, 3, 4 or more serves) and were collapsed, if necessary, when low counts were observed. 

All food purchasing and consumption behaviour indicators were entered into a multinomial logistic regression model to formally examine their relationship with FS status. Age, household income, marital status and education were established as significant sociodemographic predictors of FS status in the authors’ previous research [9]. These variables were controlled for in all models. Statistical Package for Social Sciences (SPSS) (IBM Corp. Version 20, Armonk, NY, USA) was employed to analyse the survey data. Statistical significance was set at *p* ≤ 0.05.

### 2.3. Ethics Approval

Ethics approval was granted by Edith Cowan University’s Human Research Ethics Committee (# 11118). All participants involved in this study provided informed written consent upon agreeing to complete the survey. All survey data were non-identifiable, with no individual identifier labels ever utilised. 

## 3. Results

### 3.1. Sample Demographics

The survey was completed by 1056 Australian participants (5442 invited, response rate: 19.4%). Table 1 describes the respondent group using characteristics, with the exception of gender, previously established to be associated with FS. The majority of respondents were classified as having high–marginal FS status (63.4%, *n* = 670), followed by low (19.8%, *n* = 209) and then very low (16.8%, *n* = 177). In relation to household income, respondents were most likely to indicate an income in the low (31.6 %, *n* = 334), followed by the middle (24.3 %, *n* = 257) and high (24.1%, *n* = 255) brackets (Table 1). Approximately two thirds (62.4 %, *n* = 659) of respondents were in a de facto relationship or married. Over 73% (*n* = 771) of respondents had attained some form of post-secondary education. 

### 3.2. Food Purchasing Behaviours and Food Security Status

From the 28 food purchasing indicators assessed, just under half (*n* = 11) were significantly associated with FS status (refer to Table 2 and Table 3). Of the significant indicators, five were related to “food labels” (Table 2) and six were “food product attributes” (Table 3). None of the nutrition claims were found to be significant. All models were adjusted for education, household income, marital status and age. The response rates (Tables S1–S3) and analysis of all indicators (Tables S4–S6) can be found in the Supplementary Materials.

#### 3.2.1. Food Label Indicators

Respondents with high–marginal FS status were more likely to agree that they understood the information on the back of packaging (*p* < 0.001) and that food label information was useful (*p* = 0.002), in comparison to those with low or very low FS status (Table 2). In contrast to the high–marginal FS group, low or very low FS respondents were less likely to read food labels (*p* < 0.001), be influenced by product nutrition information (*p* = 0.002) and were more likely to think there was too much information on a food packages (*p* < 0.001) (Table 2).

#### 3.2.2. Food Product Characteristics

No significant relationships were reported between FS status and cost (*p* = 0.117), Australian grown products (*p* = 0.889), those in season (*p* = 0.614), local (*p* = 0.205) or unprocessed products (*p* = 0.521). High–marginal FS respondents were more likely to rate quality (*p* = 0.010) and nutrition (*p* = 0.021) as important when compared to low or very low FS respondents (Table 3). Conversely, convenience (*p* < 0.001), organic (*p* = 0.027) and supermarket-branded products (*p* < 0.001) were more likely to be rated as important by low FS respondents than those with high–marginal FS (Table 3).

### 3.3. Food Consumption

When adjusted for sociodemographic variables, eight food groupings (bread, fruit juice, salad and vegetables, potato (not including chips), pasta, rice or noodles, poultry, nuts and seeds and water) were significantly associated with FS status (Table 4). High–marginal FS respondents were more likely to report higher consumption of salad and vegetables (*p* = 0.003), pasta, rice or noodles (*p* = 0.007) and nuts and seeds (*p* < 0.001) compared to food-insecure respondents. Very low FS respondents cited the lowest consumption of bread (*p* = 0.014), potato (*p* < 0.001) and poultry (*p* = 0.020). Food-insecure respondents reported the highest fruit juice (*p* = 0.043) intake and, conversely, the lowest water (*p* < 0.001) intake. Refer to Table S7 in the Supplementary Materials for consumption indicator response rates. When asked to rate “how healthy” their diet was, high–marginal FS respondents were twice as likely to describe their diet as healthy than very low FS respondents (*p* = 0.001). 

## 4. Discussion

This study contributes to the understanding of how food labels and other factors are associated with the purchasing decisions of a food-insecure population. These findings indicate those experiencing food insecurity are less likely to self-report understanding, using or being influenced by food labels. Similarly, Gittelsohn, Song, Anliker, Sharma and Mattingly [20] found food-insecure households in Baltimore City in the USA had the lowest label reading scores, and the procurement of healthy food was directly related to this nutrition knowledge. Interest in food labels is generally thought to be high, with approximately 70% individuals reporting taking notice of food labels in both Israel and the United Kingdom [17,18]. Similarly, the majority of our study’s respondents (53% low and very low FS to 59%—high–marginal FS) indicated they read food labels. However, Sharf, Sela, Zentner, Shoob, Shai and Stein-Zamir [19] suggested consumer understanding of these labels is low. Self-reported understanding of food labels in our study was relatively low in general (59%) but was significantly lower in the food-insecure group (48%). This significance remained even when formal education was controlled for, implying that higher educational attainment may not necessarily translate into the demonstration of greater food literacy skills in food-insecure populations. This lack of knowledge or food literacy may result in an inability to decode or apply information and may be linked to a sense of being deliberately misled, which may ultimately impact healthy food choices [28]. 

Despite nutrition labels not being rated as important, our study revealed that convenience, organic status and supermarket-branded products were seen as important by food-insecure individuals. Cost or perceived cost of healthy food is frequently cited as a significant barrier for food-insecure households [8,29,30,31,32,33], yet cost was not found to be significantly related to food insecurity in our research. A possible explanation for this is that the vast majority of respondents, regardless of FS status, considered cost an important determining factor in their purchasing decision process. Food-insecure respondents did, however, look for supermarket “home” brands or “no name” brands more than their food-secure counterparts, and these tend to be a cheaper option. Previous research has identified purchasing supermarket-branded products as a cost-saving measure employed by food-insecure households [11]. Other coping strategies identified in the literature are reducing the quality and nutritional value of food as a means of maximising the household spending power [34]. These coping strategies are reflected in the present research, where quality and nutrition were rated as less important by food-insecure respondents.

Lack of time has been cited as a barrier to obtaining and preparing healthy foods in food-insecure households in several studies [29,33,35,36]. Indeed, food-insecure respondents in our research highly valued convenience, but it is unclear how these individuals make trade-offs between convenience and price. Another product attribute considered important by food-insecure respondents in this study was organic produce. This mirrored the results from our previous research, where food-insecure interviewees indicated that organic food equated to healthy food, and this was one of the reasons cited for the perceived higher cost of nutritious food [36]. It is plausible that increased provision of food literacy education, with an emphasis on understanding food labels, identification of cost-effective and convenient healthy food options, may assist those experiencing low food security in maximising their income and diet diversity [37]. More research is warranted in this area. 

There is a growing body of evidence demonstrating food insecurity is associated with suboptimal nutritional status and poorer diet quality [38,39,40]. Certainly, food-insecure respondents in our study were more likely to perceive their diets to be “unhealthy” when compared to their food-secure counterparts. There is agreeance in the research that food insecurity is related to ongoing dietary compromise [4,6,20], but there is debate about the exact concessions made. Respondents classified as having low FS in this study reported greater or similar intakes to their high–marginal FS counterparts for many food groups. This implies, in line with the definition of low FS, that whilst these respondents may be reducing the quality or variety within the food groups, the overall volume of food consumed appears to be similar. Australian adults experiencing food insecurity in this study consumed significantly less pasta, noodles or rice, salad or other vegetables and water compared to those who were food-secure. On the other hand, fruit juice intake was higher in food-insecure households. In agreeance with our findings, several other studies have indicated food-insecure households may displace water intake with sugar sweetened beverages [39,41]. Canadian and US studies have demonstrated greater consumption of fast food, sugary drinks, fruit juice and snack foods in food-insecure populations [11,42,43,44]. Nevertheless, findings from outside of North America are less consistent, perhaps highlighting differing eating patterns and behaviours based on geographical location and culture. For example, food-insecure Pacific Island families residing in New Zealand were more likely to favour nutritionally dense foods (bread, fruit and vegetables) instead of energy dense foods (sugary drinks, ice cream and alcohol) [45]. 

Approximately 90% of all respondent groups in our study cited vegetable intakes below the recommendation of five serves [27], results similar to Australian national survey findings [46]. Despite the overall low intake of vegetables by participants in our study, the food-insecure group still demonstrated a significantly lower consumption rate in comparison to those who were food-secure. Compared to the results of a New Zealand study, our findings also suggest food-insecure households may be prepared to reduce spending on vegetables or bread but not meat products, with the exception of poultry. Furthermore, poultry and nuts and seeds are perceived as ‘luxury’ or expensive items in Australia [38], and this may explain limited intake of these foods in food-insecure households.

Although this study utilised a large, representative sample size, there are several limitations that need to be considered when interpreting the results. Firstly, self-reported research is subject to various disadvantages, including social desirability bias [47]. This study only investigated the self-reported understanding and use of food labels. Observational research has found consumer interaction with food labels was considerably lower than self-reported usage and understanding [48]. Therefore, the actual food label behaviours of the respondents may be different from those cited in this study’s survey. Secondly, the dietary consumption tool used in this study did allow for comparison to the Australian dietary guidelines; however, changes in food quality and variety over time [5,6], fast food intake [8] or cyclical eating patterns [49] were not captured. These are potentially important dietary considerations for food-insecure populations. Thirdly, aspects not considered in this study were the impact of Aboriginal and Torres Strait Islander status or geographical remoteness. This may be important as both the aforementioned aspects are considered noteworthy social determinants of FS in Australia [50,51,52], and therefore, generalising these results across all sub populations is cautioned.

## 5. Conclusions

This research indicates food-insecure respondents are less likely to self-report understanding, using or being influenced by food labels when making purchasing decisions. However, factors such as convenience, super-market-branded and organic status were viewed as important by this subpopulation. Food-insecure respondents in this study were more likely to consider their diet “unhealthy”, and there were variances in reported dietary intake when compared to their food-secure counterparts. It is possible that factors other than the income or financial constraints outlined in this study may have an impact on the dietary intake of food-insecure people, but more research is needed to support this theory. 

## Figures and Tables

**Table 1:** Characteristics of survey respondents compared by food security status.

Independent Variable	Category	Overall Significance ^b^	Total *n* (% of *n* = 1056)	High–Marginal Food Security *n* = 670 (63.4%)	Low Food Security *n* = 209 (19.8%)	Very Low Food Security *n* = 177 (16.8%)
Gender		0.256				
	Male		329 (31.2%)	225 (33.6%)	56 (26.8%)	48 (27.1%)
	Female		727 (68.8%)	445 (66.4%)	153 (73.2%)	129 (72.9%)
Age (years)		<0.001 **				
	19–24		81 (7.7%)	53 (7.9%)	14 (6.7%)	14 (7.9%)
	25–34		195 (18.5%)	102 (15.3%)	52 (24.9%)	41 (23.2%)
	35–44		212 (20.1%)	128 (19.1%)	44 (21.1%)	40 (22.6%)
	45–54		219 (20.7%)	141 (21.0%)	42 (20.1%)	36 (20.3%)
	55–64		181 (17.1%)	120 (17.9%)	30 (14.4%)	31 (17.5%)
	65–84		168 (15.9%)	126 (18.8%)	27 (12.8%)	15 (8.5%)
Marital status		0.006 **				
	Widowed		37 (3.5%)	27 (4.0%)	5 (2.4%)	5 (2.8%)
	Divorced/Separated		108 (10.2%)	56 (8.4%)	21 (10.0%)	31 (17.5%)
	Married/De facto		659 (62.4%)	440 (65.7%)	127 (60.8%)	92 (52.0%)
	Single		252 (23.9%)	147 (21.9%)	56 (26.8%)	49 (27.7%)
Household income ($AUD)		<0.001 **				
	Very low (<$18,000)		35 (3.3%)	16 (2.4%)	6 (2.9%)	13 (7.3%)
	Low ($18,001–37,000)		334 (31.6%)	181 (27.0%)	78 (37.3%)	75 (42.4%)
	Middle ($37,001–87,000)		257 (24.3%)	166 (24.8%)	45 (21.5%)	46 (26.0%)
	High ($87,001–180,000)		255 (24.1%)	180 (26.9%)	51 (24.5%)	24 (13.6%)
	Very high (>$180,000)		75 (7.1%)	64 (9.5%)	8 (3.8%)	3 (1.7 %)
	Did not answer		100 (9.6%)	63 (9.4%)	21 (10.0%)	16 (9.0%)
Education completed		<0.001 **				
	Secondary or less		285 (27.0%)	161 (24.1%)	66 (31.6%)	58 (32.7%)
	Vocational ^a^		401 (38.0%)	240 (35.8%)	89 (42.6%)	72 (40.7%)
	University		370 (35.0%)	269 (40.1%)	54 (25.8%)	47 (26.6%)

^a^ Vocational considered to be post-secondary; ^b^ Multinomial logistic regression was used to establish significance; ** *p*-value < 0.01.

**Table 2:** Significant food label and single consumption indicators. ^a^

Outcome	Category	Overall Significance ^a^	Post Hoc Analysis					
			High–Marginal vs. Very Low Food Security		High–Marginal vs. Low Food Security		Low Food Security Vs. Very Low Food Security	
			OR (95% CI)	*p*-Value	OR (95% CI)	*p*-Value	OR (95% CI)	*p*-Value
I understand the information provided on the back of food packages	Agree	<0.001 **	2.83 (1.72, 4.66)	<0.001 **	1.46 (0.86, 2.49)	0.146	1.93 (1.07, 3.51)	0.030 *
	Neither agree nor disagree		1.67 (0.98, 2.86)	0.062	0.73 (0.42, 1.27)	0.261	2.29 (1.23, 4.26)	0.009 **
	Disagree		1.00 (ref)		1.00 (ref)		1.00 (ref)	
The ingredients and nutritional information on the back of the package does not influence my purchasing decisions	Agree	0.002 **	0.82 (0.52, 1.30)	0.399	0.52 (0.35, 0.78)	0.001 **	1.58 (0.94, 2.65)	0.088
	Neither agree nor disagree		0.60 (0.40, 0.90)	0.013 *	0.58 (0.39, 0.86)	0.007 **	1.02 (0.63, 1.66)	0.929
	Disagree		1.00 (ref)		1.00 (ref)		1.00 (ref)	
The nutrition information offers useful information about the product	Agree	0.002 **	3.26 (1.67, 6.37)	0.001 **	1.59 (0.77, 3.27)	0.208	2.05 (0.95, 4.42)	0.066
	Neither agree nor disagree		2.04 (1.01, 4.11)	0.046	1.08 (0.51, 2.28)	0.843	1.89 (0.85, 4.12)	0.117
	Disagree		1.00 (ref)		1.00 (ref)		1.00 (ref)	
There is too much nutritional information on food packaging	Agree	<0.001 **	0.44 (0.28, 0.70)	<0.001 **	0.57 (0.36, 0.92)	0.020 *	0.77 (0.44, 1.34)	0.352
	Neither agree nor disagree		1.02 (0.68, 1.54)	0.924	0.52 (0.36, 0.75)	0.001 **	1.95 (1.21, 3.15)	0.007 **
	Disagree		1.00 (ref)		1.00 (ref)		1.00 (ref)	
I never read the nutritional information and ingredients on food packages	Agree	<0.001 **	0.48 (0.29, 0.79)	0.004 **	0.54 (0.34, 0.87)	0.011 *	0.86 (0.50, 1.58)	0.678
	Neither agree nor disagree		0.44 (0.29, 0.66)	<0.001 **	0.33 (0.23, 0.48)	0.001 **	1.32 (0.83, 2.09)	0.245
	Disagree		1.00 (ref)		1.00 (ref)		1.00 (ref)	
How healthy would you say your diet was?	Healthy	0.001 **	2.17 (1.44, 3.27)	<0.001	1.31 (0.87, 1.95)	0.195	1.66 (1.04, 2.67)	0.034 *
	Unhealthy		1.00 (ref)		1.00 (ref)		1.00 (ref)	

^a^ Multinomial logistic regression model was adjusted for sociodemographic variables (age, household income, education and marital status). * *p*-value < 0.05; ** *p*-value < 0.01; OR = odds ratio; CI = confidence interval; 1.00 (ref) = reference level.

**Table 3:** Significant food product attributes. ^a^

Outcome	Category	Overall Significance ^a^	Post Hoc Analysis					
			High–Marginal vs. Very Low Food Security		High–Marginal vs. Low Food Security		Low Food Security vs. Very Low Food Security	
			OR (95% CI)	*p*-Value	OR (95% CI)	*p*-Value	OR (95% CI)	*p*-Value
Nutrition	Important	0.021 *	1.00 (ref)		1.00 (ref)		1.00 (ref)	
	Neither important nor unimportant		0.49 (0.30, 0.81)	0.005 **	0.53 (0.34, 0.84)	0.006 **	0.93 (0.54, 1.59)	0.790
	Unimportant		0.73 (0.28, 1.91)	0.531	0.82 (0.33, 2.05)	0.667	0.90 (0.30,2.69)	0.853
Quality	Important	0.010 *	1.00 (ref)		1.00 (ref)		1.00 (ref)	
	Neither important nor unimportant		0.40 (0.20, 0.81)	0.011 *	0.40 (0.21, 0.76)	0.005 **	1.00 (0.47, 2.11)	0.994
	Unimportant		0.36 (0.10, 1.28)	0.115	0.383 (0.12, 1.28)	0.119	0.94 (0.25, 3.53)	0.932
Organic	Important	0.027 *	0.65 (0.42, 1.01)	0.056	0.55 (0.36, 0.83)	0.005 **	1.19 (0.70, 2.00)	0.520
	Neither important nor unimportant		1.01 (0.66, 1.56)	0.963	0.73 (0.49, 1.08)	0.116	1.39 (0.83, 2.33)	0.205
	Unimportant		1.00 (ref)		1.00 (ref)		1.00 (ref)	
Raw food (natural state)	Important	0.024 *	1.119 (0.71, 1.77)	0.632	0.57 (0.35, 0.93)	0.023 *	1.96 (1.10, 3.47)	0.022 *
	Neither important nor unimportant		1.60 (0.98, 2.62)	0.059	0.77 (0.47, 1.273)	0.309	2.08 (1.13, 3.82)	0.018 *
	Unimportant		1.00 (ref)		1.00 (ref)		1.00 (ref)	
Convenience (pre-packaged to save time) e.g., pre-cut vegetables, pre-marinated meats, bottle sauces	Important	<0.001 **	0.53 (0.34, 0.83)	0.005 **	0.40 (0.27, 0.61)	<0.001 **	1.31 (0.77, 2.22)	0.325
	Neither important nor unimportant		0.55 (0.36, 0.85)	0.007 **	0.52 (0.35, 0.79)	0.002 **	1.06 (0.63, 1.78)	0.833
	Unimportant		1.00 (ref)		1.00 (ref)		1.00 (ref)	
Supermarket branded (home brand, Coles Select)	Important	<0.001 **	0.37 (0.23, 0.60)	<0.001 **	0.214 (0.13, 0.35)	<0.001 **	1.74 (0.97, 3.12)	0.066
	Neither important nor unimportant		0.83 (0.53, 1.28)	0.391	0.475 (0.30, 0.74)	0.001 **	1.74 (0.99, 3.05)	0.053
	Unimportant		1.00 (ref)		1.00 (ref)		1.00 (ref)	

^a^ Multinomial logistic regression model was adjusted for sociodemographic variables (age, household income, education and marital status). * *p*-value < 0.05; ** *p*-value < 0.01; OR = odds ratio; CI = confidence interval; 1.00 (ref) = reference level.

**Table 4:** Consumption behaviours related to food and beverages (with example serve sizes ^a^ given) by food security status. ^b^

Question		Serves	*n* (%)	Overall Significance	Post-Hoc Analysis					
					High–Marginal vs. Very Low Food Security		High–Marginal vs. Low Food Security		Low Food Security vs. Very Low Food Security	
					OR (95% CI)	*p*-Value	OR (95% CI)	*p*-Value	OR (95% CI)	*p*-Value
On a Typical Day, How Many Serves of the Following Foods Would You Eat?										
Breakfast cereals	2/3 cup breakfast cereals, cooked oats	0	200 (19.5%)	0.525	1.00 (Ref)		1.00 (Ref)		1.00 (Ref)	
	2 wheat-biscuits	1	736 (71.7%)		1.41 (0.91, 2.19)	0.127	1.13 (0.74, 1.73)	0.566	1.25 (0.74, 2.09)	0.403
		2 or more	90 (8.8%)		1.49 (0.7, 3.17)	0.296	0.9 (0.47, 1.71)	0.739	1.67 (0.71, 3.89)	0.238
Milk, yoghurt, cheese and dairy alternatives	1 cup of milk or soy milk	0	76 (7.4%)	0.496	1.00 (Ref)		1.00 (Ref)		1.00 (Ref)	
	2 slices of cheese	1	568 (55.4%)		1.39 (0.71, 2.72)	0.332	1.24 (0.66, 2.32)	0.501	1.12 (0.52, 2.4)	0.766
	1 tub of yoghurt	2	288 (28.1%)		1.67 (0.81, 3.42)	0.163	1.66 (0.85, 3.27)	0.139	1 (0.44, 2.29)	0.998
		3 or more	94 (9.2%)		1.86 (0.77, 4.46)	0.166	1.16 (0.53, 2.53)	0.714	1.6 (0.6, 4.32)	0.350
Bread	1 slice of bread	0	71 (6.9%)	0.014 *	1.00 (Ref)		1.00 (Ref)		1.00 (Ref)	
	1 crumpet or English muffin	1	414 (40.4%)		1.85 (0.95, 3.6)	0.069	0.85 (0.41, 1.77)	0.669	2.17 (0.96, 4.93)	0.064
		2	428 (41.7%)		3.05 (1.55, 5.99)	0.001 **	1.17 (0.56, 2.44)	0.674	2.6 (1.13, 6.01)	0.025 *
		3 or more	113 (11%)		2.64 (1.15, 6.05)	0.022 *	0.79 (0.35, 1.81)	0.579	3.34 (1.25, 8.92)	0.016 *
Fruit (not including juice)	1 medium banana, apple or orange	0	56 (5.5%)	0.080	1.00 (Ref)		1.00 (Ref)		1.00 (Ref)	
	2 small kiwi fruit, apricots or plums	1	480 (46.8%)		2.52 (1.24, 5.09)	0.010 **	1.14 (0.53, 2.47)	0.735	2.2 (0.95, 5.13)	0.067
	1 cup canned fruit	2	327 (31.9%)		3.45 (1.65, 7.21)	0.001 **	1.39 (0.63, 3.06)	0.416	2.48 (1.03, 6.01)	0.043 *
	A handful of dried fruit (e.g., 4 apricot halves)	3 or more	163 (15.9%)		2.6 (1.17, 5.8)	0.020 **	1.32 (0.56, 3.1)	0.525	1.97 (0.75, 5.18)	0.169
Fruit juice	1 cup fruit juice	0	288 (28.1%)	0.043 *	1.00 (Ref)		1.00 (Ref)		1.00 (Ref)	
		1	598 (58.3%)		1.39 (0.92, 2.11)	0.117	0.87 (0.58, 1.28)	0.472	1.61 (0.98, 2.64)	0.060
		2 or more	140 (13.6%)		0.78 (0.44, 1.36)	0.374	0.54 (0.32, 0.93)	0.027 *	1.43 (0.75, 2.72)	0.281
Salad and vegetables (not including potato)	1 cup salad vegetables (e.g., lettuce, cucumber, tomato)½ cup cooked or canned vegetables	0	44 (4.3%)	0.003 **	1.00 (Ref)		1.00 (Ref)		1.00 (Ref)	
		1	463 (45.1%)		4.08 (1.74, 9.56)	0.001 **	3.13 (1.38, 7.11)	0.006 **	1.3 (0.55, 3.08)	0.547
		2	292 (28.5%)		5.03 (2.09, 12.08)	<0.001 **	4.6 (1.97, 10.75)	<0.001 **	1.09 (0.44, 2.7)	0.847
		3	148 (14.4%)		3.81 (1.5, 9.69)	0.005 **	3.32 (1.36, 8.08)	0.008 **	1.15 (0.44, 3.03)	0.778
		4 or more	79 (7.7%)		4.58 (1.61, 13.04)	0.004 **	6.18 (2.17, 17.57)	0.001 **	0.74 (0.23, 2.4)	0.619
Potato (not including chips)	½ medium potato	0	80 (7.8%)	<0.001 **	1.00 (Ref)		1.00 (Ref)		1.00 (Ref)	
	½ cup mashed potato	1	763 (74.4%)		3.88 (2.09, 7.19)	<0.001 **	2.46 (1.35, 4.51)	0.003 **	1.57 (0.81, 3.07)	0.185
		2 or more	183 (17.8%)		2.78 (1.37, 5.66)	0.005 **	0.64 (0.33, 1.24)	0.185	1.63 (0.75, 3.54)	0.218
Pasta, rice, or noodles	½ cup cooked pasta or rice, noodles	0	74 (7.2%)	0.007 **	1.00 (Ref)		1.00 (Ref)		1.00 (Ref)	
		1	739 (72%)		2.89 (1.5, 5.59)	0.002 **	2.5 (1.35, 4.61)	0.003 **	1.16 (0.57, 2.35)	0.682
		2 or more	213 (20.8%)		2.63 (1.26, 5.5)	0.010 **	1.99 (1.01, 3.92)	0.046 *	1.32 (0.6, 2.93)	0.491
Meat alternatives	1 cup baked beans, cooked legumes or tofu	0	121 (11.8%)	0.151	1.00 (Ref)		1.00 (Ref)		1.00 (Ref)	
		1	764 (74.5%)		1.8 (1.06, 3.05)	0.031 *	1.65 (1.01, 2.71)	0.046 *	1.09 (0.6, 1.97)	0.786
	2 large eggs	2 or more	141 (13.7%)		1.53 (0.78, 2.99)	0.218	1.67 (0.88, 3.16)	0.119	0.92 (0.42, 1.99)	0.825
Fish	A cooked fish fillet about the size of an open hand (100 g)	0	141 (13.7%)	0.272	1.00 (Ref)		1.00 (Ref)		1.00 (Ref)	
		1	747 (72.8%)		1.47 (0.9, 2.41)	0.128	0.9 (0.54, 1.5)	0.694	1.63 (0.89, 2.96)	0.111
	One small can of fish (100 g)	2 or more	138 (13.5%)		1.25 (0.64, 2.45)	0.505	0.65 (0.35, 1.22)	0.179	1.93 (0.89, 4.19)	0.095
Poultry	Cooked lean poultry such as chicken or turkey, about the size of an open hand (80 g)	0	69 (6.7%)	0.020 *	1.00 (Ref)		1.00 (Ref)		1.00 (Ref)	
		1	758 (73.9%)		2.25 (1.19, 4.27)	0.013 *	1.1 (0.53, 2.28)	0.798	2.05 (0.91, 4.6)	0.084
		2 or more	199 (19.4%)		1.96 (0.95, 4.05)	0.069	0.68 (0.31, 1.48)	0.334	2.87 (1.18, 6.98)	0.020 *
Red meat	Cooked lean meat, about the size of a deck of playing cards (65 g)	0	95 (9.3%)	0.611	1.00 (Ref)		1.00 (Ref)		1.00 (Ref)	
		1	736 (71.7%)		1.42 (0.78, 2.58)	0.249	1.1 (0.61, 2)	0.745	1.29 (0.63, 2.63)	0.489
		2 or more	195 (19%)		1.6 (0.79, 3.21)	0.189	0.93 (0.48, 1.81)	0.837	1.71 (0.75, 3.89)	0.199
Nuts and seeds	A handful of nuts /seeds	0	174 (17%)	0.001 **	1.00 (Ref)		1.00 (Ref)		1.00 (Ref)	
		1	662 (64.5%)		2.3 (1.45, 3.64)	<0.001 **	1.23 (0.78, 1.95)	0.370	1.86 (1.08, 3.2)	0.025 *
		2 or more	190 (18.5%)		1.33 (0.76, 2.34)	0.322	0.73 (0.42, 1.27)	0.266	1.82 (0.95, 3.48)	0.071
Savoury snacks	2 slices of processed meat	0	157 (15.3%)	0.396	1.00 (Ref)		1.00 (Ref)		1.00 (Ref)	
	12 hot chips	1	676 (65.9%)		1.61 (0.99, 2.6)	0.054	1.02 (0.63, 1.65)	0.928	1.57 (0.88, 2.81)	0.127
	½ small packet of crisps (20 g)	2 or more	193 (18.8%)		1.29 (0.72, 2.33)	0.392	0.94 (0.53, 1.67)	0.835	1.37 (0.68, 2.76)	0.371
Sweet snacks	2 scoops ice cream	0	107 (10.4%)	0.165	1.00 (Ref)		1.00 (Ref)		1.00 (Ref)	
	1 doughnut, slice of cake, muffin	1	718 (70%)		1.97 (1.14, 3.4)	0.015 *	1.51 (0.89, 2.58)	0.130	1.3 (0.69, 2.44)	0.412
	½ regular bar of chocolate (25 g)	2 or more	201 (19.6%)		1.92 (1.01, 3.62)	0.045 *	1.49 (0.8, 2.77)	0.207	1.29 (0.62, 2.67)	0.500
	2–3 biscuits									
Water (including tea and coffee)	1 cup (250 mL)	0	38 (3.7%)	<0.001 **	1.00 (Ref)		1.00 (Ref)		1.00 (Ref)	
		1	185 (18%)		5.41 (2, 14.61)	0.001 **	2.8 (1.09, 7.18)	0.033 *	1.94 (0.74, 5.03)	0.175
		2	118 (11.5%)		5.83 (2.05, 16.59)	0.001 **	3.69 (1.36, 10.01)	0.010 **	1.58 (0.56, 4.45)	0.386
		3	140 (13.6%)		5.41 (1.95, 15.05)	0.001 **	4.19 (1.55, 11.36)	0.005 **	1.29 (0.47, 3.57)	0.623
		4 or more	545 (53.1%)		10.63 (4.12, 27.4)	<0.001 **	5.07 (2.05, 12.54)	<0.001 **	2.1 (0.85, 5.15)	0.107
Additional drinks (not including alcohol)	1 can of soft drink (375 mL)	0	255 (24.9%)	0.076	1.00 (Ref)		1.00 (Ref)		1.00 (Ref)	
	2 cups of cordial (500 mL)	1	564 (55%)		1.16 (0.75, 1.81)	0.505	0.87 (0.57, 1.32)	0.508	1.34 (0.79, 2.28)	0.282
	1 can energy drink (330 mL)	2	143 (13.9%)		0.79 (0.44, 1.43)	0.437	0.68 (0.39, 1.19)	0.174	1.16 (0.58, 2.32)	0.666
	2 cups of Sports drink (500 mL)	3 or more	64 (6.2%)		0.41 (0.2, 0.87)	0.019 *	0.47 (0.23, 0.97)	0.042 *	0.87 (0.38, 2)	0.750
Alcohol	30 mL spirits	0	270 (26.3%)	0.202	1.00 (Ref)		1.00 (Ref)		1.00 (Ref)	
	60 mL fortified wine	1	367 (35.8%)		1.33 (0.86, 2.08)	0.204	1.2 (0.79, 1.83)	0.390	1.11 (0.66, 1.86)	0.698
	100 mL wine	2	214 (20.9%)		1.4 (0.84, 2.35)	0.198	1.34 (0.82, 2.19)	0.239	1.05 (0.57, 1.92)	0.885
	425 mL light beer	3	81 (7.9%)		1.43 (0.68, 3.01)	0.341	1.12 (0.58, 2.18)	0.731	1.28 (0.54, 3.01)	0.575
	285 mL regular beer	4 or more	94 (9.2%)		3.56 (1.49, 8.51)	0.004 **	1.26 (0.69, 2.32)	0.455	2.82 (1.09, 7.34)	0.033
	Small bottle of premix drink or “alco-pop” (300 mL)									
								

^a^ Serves as defined by the Australian Dietary Guidelines ^b^ Multinomial logistic regression model was adjusted for sociodemographic variables (age, household income, education and marital status). * *p*-value < 0.05; ** *p*-value < 0.01; OR = odds ratio; CI = confidence interval; 1.00 (ref) = reference level.

## Supplementary Materials

The following are available online at https://www.mdpi.com/2072-6643/11/4/828/s1, Table S1: Response, by three categories of food security, frequencies and proportions for a single consumption and food label indicators, Table S2: Response, by three categories of food security, frequencies and proportions for nutrition claim indicators, Table S3: Response, by three categories of food security frequencies and proportions for product attribute indicators, Table S4: Response, by three categories of food security *p*-values and odds ratios of single consumption and food label indicators, Table S5: Response, by three categories of food security *p*-values and odds ratios of nutrition claim indicators, Table S6: Response, by three categories of food security *p*-values and odds ratios of product attribute indicators, Table S7: Response, by three categories of food security, frequencies and proportions for consumption behaviours.
