# Supplementary material for: Food-Insecure Household’s Self-Reported Perceptions of Food Labels, Product Attributes and Consumption Behaviours
Source: Nutrients. 2019 Apr 12;11(4):828. doi: 10.3390/nu11040828 (PMC6521285; doi:10.3390/nu11040828)
Supplement: Supplementary file 1 [file nutrients-11-00828-s001.pdf]

nutrients

MDPI

Table S1: Response, by three categories of food security, frequencies and proportions for a single consumption and food label indicators

Outcome	Category	High-Marginal Food Security	Low Food Security	Very Low Food Security
I read the ingredients and nutrition information on the back of the food package	Agree	392 (59%)	110 (53%)	94 (53%)
	Neither agree nor disagree	161 (24%)	67 (23%)	48 (27%)
	Disagree	115 (17%)	30 (14%)	35 (20%)
I understand the information provided on the back of food packages	Agree	439 (66%)	100 (48%)	82 (46%)
	Neither agree nor disagree	168 (25%)	82 (40%)	55 (31%)
	Disagree	61 (9%)	25 (12%)	40 (23%)
I take notice of the nutritional claims on the front of food packaging. e.g. low fat, high calcium, high fibre, diet, lite	Agree	394 (59%)	108 (52%)	89 (50%)
	Neither agree nor disagree	150 (22%)	65 (31%)	51 (29%)
	Disagree	124 (19%)	34 (16%)	37 (21%)
I still read the nutritional information and ingredients on the back of the package if there is a claim on the front	Agree	413 (62%)	112 (54%)	97 (55%)
	Neither agree nor disagree	144 (21%)	58 (28%)	46 (26%)
	Disagree	111 (17%)	37 (18%)	34 (19%)
I prefer to buy food that carries a nutritional claim on the front of the package	Agree	165 (25%)	58 (28%)	49 (28%)
	Neither agree nor disagree	320 (48%)	106 (51%)	77 (44%)
	Disagree	183 (27%)	43 (21%)	51 (29%)
The ingredients and nutritional information on the back of the package does not influence my purchasing decisions	Agree	160 (27%)	68 (33%)	41 (23%)
	Neither agree nor disagree	178 (27%)	70 (34%)	69 (39%)
	Disagree	330 (49%)	69 (33%)	67 (38%)
The nutrition information offers useful information about the product	Agree	475 (71%)	124 (60%)	98 (55%)
	Neither agree nor disagree	166 (25%)	70 (34%)	59 (34%)
	Disagree	27 (4%)	13 (6%)	20 (11%)
There is too much nutritional information on food packaging	Agree	109 (16%)	38 (19%)	50 (28%)
	Neither agree nor disagree	241 (36%)	102 (49%)	58 (33%)
	Disagree	318 (48%)	67 (32%)	69 (39%)
I never read the nutritional information and ingredients on food packages	Agree	81 (12%)	33 (16%)	32 (18%)
	Neither agree nor disagree	118 (18%)	76 (37%)	54 (31%)
	Disagree	469 (70%)	98 (47%)	91 (51%)
How healthy would you say your diet was?	Healthy	540 (83%)	154 (76%)	111 (65%)
	Unhealthy	109 (17%)	50 (25%)	60 (35%)

Table S2: Response, by three categories of food security, frequencies and proportions for nutrition claim indicators

Outcome	Category	High-Marginal Food Security	Low Food Security	Very Low Food Security
Rate the importance of the nutritional claim:	Important	381 (57%)	123 (59%)	94 (53%)
Low calorie (kilojoule)	Neither important nor unimportant	186 (28%)	56 (27%)	60 (34%)
	Unimportant	101 (15%)	28 (14%)	23 (13%)
Rate the importance of the nutritional claim:	Important	337 (50%)	118 (57%)	98 (55%)
High protein	Neither important nor unimportant	235 (35%)	69 (33%)	64 (36%)
	Unimportant	96 (14%)	20 (20%)	15 (9%)
Rate the importance of the nutritional claim:	Important	470 (70%)	143 (69%)	122 (69%)
Low saturated fats	Neither important nor unimportant	132 (20%)	46 (22%)	41 (23%)
	Unimportant	66 (10%)	18 (9%)	14 (8%)
Rate the importance of the nutritional claim:	Important	309 (46%)	107 (52%)	96 (54%)
Low carbohydrates	Neither important nor unimportant	252 (38%)	70 (34%)	62 (35%)
	Unimportant	107 (16%)	30 (15%)	19 (11%)
Rate the importance of the nutritional claim:	Important	443 (66%)	134 (65%)	109 (62%)
Low sodium	Neither important nor unimportant	162 (24%)	51 (0.25%)	51 (29%)
	Unimportant	63 (9%)	22 (11%)	17 (10%)
Rate the importance of the nutritional claim:	Important	486 (73%)	150 (73%)	125 (71%)
Low sugar	Neither important nor unimportant	135 (20%)	38 (18%)	36 (20%)
	Unimportant	47 (7%)	19 (9%)	16 (9%)
Rate the importance of the nutritional claim:	Important	329 (49%)	109 (53%)	91 (51%)
Low glycemic index	Neither important nor unimportant	235 (35%)	78 (37%)	63 (36%)
	Unimportant	104 (16%)	20 (10%)	23 (13%)
Rate the importance of the nutritional claim:	Important	429 (64%)	138 (66%)	117 (66%)
Low preservatives	Neither important nor unimportant	180 (27%)	59 (29%)	49 (28%)
	Unimportant	59 (9%)	10 (5%)	11 (6%)

Table S3: Response, by three categories of food security frequencies and proportions for product attribute indicators

Outcome	Category	High-Marginal Food Security	Low Food Security	Very Low Food Security
Nutrition	Important	583 (87%)	161 (79%)	138 (78%)
	Neither important nor unimportant	66 (10%)	37 (18%)	32 (18%)
	Unimportant	18 (3%)	7 (3%)	7 (4%)
Quality	Important	633 (95%)	181 (88%)	155 (87%)
	Neither important nor unimportant	27 (4%)	19 (9%)	17 (10%)
	Unimportant	7 (1%)	5 (3%)	5 (3%)
Cost	Important	561 (84%)	181 (88%)	159 (90%)
	Neither important nor unimportant	84 (13%)	17 (8%)	12 (7%)
	Unimportant	22 (3%)	7 (4%)	6 (3%)
In season	Important	478 (72%)	141 (69%)	129 (73%)
	Neither important nor unimportant	150 (23%)	47 (23%)	37 (21%)
	Unimportant	39 (5%)	17 (8%)	11 (6%)
Local products	Important	454 (68%)	133 (65%)	117 (66%)
	Neither important nor unimportant	172 (26%)	54 (26%)	40 (23%)
	Unimportant	41 (6%)	18 (9%)	20 (11%)
Organic	Important	170 (26%)	69 (34%)	60 (34%)
	Neither important nor unimportant	254 (38%)	81 (40%)	62 (35%)
	Unimportant	243 (36%)	55 (27%)	55 (31%)
Raw food (natural state)	Important	297 (45%)	104 (51%)	81 (46%)
	Neither important nor unimportant	241 (36%)	72 (35%)	54 (30%)
	Unimportant	129 (19%)	29 (14%)	42 (24%)
Unprocessed	Important	356 (54%)	108 (53%)	94 (53%)
	Neither important nor unimportant	229 (34%)	74 (36%)	54 (31%)
	Unimportant	82 (12%)	23 (11%)	29 (16%)
Convenience (pre-packaged to save time) e.g. pre-cut vegetables, pre-marinated meats, bottle sauces	Important	176 (26%)	80 (39%)	60 (34%)
	Neither important nor unimportant	205 (31%)	74 (36%)	69 (39%)
	Unimportant	286 (43%)	51 (25%)	48 (27%)

Outcome	Category	High-Marginal Food Security	Low Food Security	Very Low Food Security
Australian grown	Important	504 (76%)	153 (75%)	124 (70%)
	Neither important nor unimportant	120 (18%)	36 (18%)	37 (21%)
	Unimportant	43 (6%)	16 (8%)	16 (9%)
Supermarket branded (homebrand, Coles Select)	Important	111 (17%)	75 (37%)	60 (34%)
	Neither important nor unimportant	325 (49%)	99 (49%)	75 (43%)
	Unimportant	231 (35%)	31 (15%)	42 (24%)

Table S4: Response, by three categories of food security p-values and odds ratios of single consumption and food label indicators

Outcome	Category	Overall Significance [a]	Post hoc analysis					
			High-Marginal vs. Very Low Food Security		High-Marginal vs. Low Food Security		Low Food Security vs. Very Low Food Security	
			**OR (95% CI)**	***p*-value**	**OR (95% CI)**	***p*-value**	**OR (95% CI)**	***p*-value**
I read the ingredients and nutrition information on the back of the food package		0.089						
	Agree		1.00 (0.63,1.61)	0.992	0.76 (0.48,1.22)	0.263	1.31 (0.74,2.33)	0.355
	Neither agree nor disagree		0.75 (0.44,1.27)	0.286	0.516 (0.31, 0.86)	0.012*	1.45 (0.77,2.72)	0.248
	Disagree		1.00 (ref)		1.00 (ref)		1.00 (ref)	
I understand the information provided on the back of food packages		<0.001**						
	Agree		2.83(1.72, 4.66)	<0.001**	1.46 (0.86, 2.49)	0.146	1.93(1.07, 3.51)	0.030*
	Neither agree nor disagree		1.67 (0.98, 2.86)	0.062	0.73 (0.42,1.27)	0.261	2.29(1.23, 4.26)	0.009**
	Disagree		1.00 (ref)		1.00 (ref)		1.00 (ref)	
I take notice of the nutritional claims on the front of food packaging. e.g. low fat, high calcium, high fibre, diet, lite		0.216						
	Agree		1.189 (0.75, 1.89)	0.464	0.91 (0.58,1.42)	0.669	1.311 (0.75,2.29)	0.339
	Neither agree nor disagree		0.857 (0.51, 1.44)	0.559	0.64 (0.39,1.05)	0.074	1.345 (0.73,2.47)	0.338
	Disagree		1.00 (ref)		1.00 (ref)		1.00 (ref)	
I still read the nutritional information and ingredients on the back of the package if there is a claim on the front		0.313						
	Agree		1.079 (0.67,1.74)	0.754	0.99 (0.63,1.55)	0.973	1.088 (0.623, 1.90)	0.767
	Neither agree nor disagree		0.791 (0.457, 1.37)	0.402	0.68 (0.41,1.13)	0.139	1.161 (0.619, 2.18)	0.642
	Disagree		1.00 (ref)		1.00 (ref)		1.00 (ref)	
I prefer to buy food that carries a nutritional claim on the front of the package		0.530						
	Agree		0.817 (0.506,1.32)	0.408	0.58 (0.36,0.92)	0.021*	1.421 (0.80,2.52)	0.230
	Neither agree nor disagree		1.258 (0.821, 1.93)	0.292	0.71 (0.47,1.08)	0.113	1.764 (1.05,2.96)	0.031*
	Disagree		1.00 (ref)		1.00 (ref)		1.00 (ref)	
The ingredients and nutritional information on the back of the package does not influence my purchasing decisions		0.002**						
	Agree		0.82 (0.52, 1.30)	0.399	0.52 (0.35, 0.78)	0.001**	1.58 (0.94, 2.65)	0.088
	Neither agree nor disagree		0.60 (0.40, 0.90)	0.013*	0.58 (0.39, 0.86)	0.007**	1.02 (0.63, 1.66)	0.929
	Disagree		1.00 (ref)		1.00 (ref)		1.00 (ref)	

**Outcome**	**Category**	**Overall Significance** [a]	**Post hoc analysis**					
			**High-Marginal vs. Very Low Food Security**		**High-Marginal vs. Low Food Security**		**Low Food Security vs. Very Low Food Security**	
			**OR (95% CI)**	***p*-value**	**OR (95% CI)**	**OR (95% CI)**	***p*-value**	**OR (95% CI)**
The nutrition information offers useful information about the product		0.002**						
	Agree		3.26 (1.67, 6.37)	0.001**	1.59 (0.77, 3.27)	0.208	2.05 (0.95, 4.42)	0.066
	Neither agree nor disagree		2.04 (1.01, 4.11)	0.046*	1.08 (0.51, 2.28)	0.843	1.89 (0.85, 4.12)	0.117
	Disagree		1.00 (ref)		1.00 (ref)		1.00 (ref)	
There is too much nutritional information on food packaging		<0.001**						
	Agree		0.44 (0.28, 0.70)	<0.001**	0.57 (0.36, 0.92)	0.020*	0.77 (0.44, 1.34)	0.352
	Neither agree nor disagree		1.02 (0.68, 1.54)	0.924	0.52 (0.36, 0.75)	0.001**	1.95 (1.21, 3.15)	0.007**
	Disagree		1.00 (ref)		1.00 (ref)		1.00 (ref)	
I never read the nutritional information and ingredients on food packages		<0.001**						
	Agree		0.48 (0.29, 0.79)	0.004**	0.54 (0.34, 0.87)	0.011*	0.86 (0.50, 1.58)	0.678
	Neither agree nor disagree		0.44 (0.29, 0.66)	<0.001**	0.33 (0.23, 0.48)	0.001**	1.32 (0.83, 2.09)	0.245
	Disagree		1.00 (ref)		1.00 (ref)		1.00 (ref)	
How healthy would you say your diet was?		0.001**						
	Healthy		2.17 (1.44, 3.27)	<0.001**	1.31 (0.87, 1.95)	0.195	1.66 (1.04, 2.67)	0.034*
	Unhealthy		1.00 (ref)		1.00 (ref)		1.00 (ref)	

[a] Multinomial logistic regression model was adjusted for socio-demographics variables (age, household income, education and marital status)

* p-value < 0.05; ** p-value < 0.01; OR = odds ratio; CI = confidence interval; 1.00 (ref) = reference level

Table S5: Response, by three categories of food security p-values and odds ratios of nutrition claim indicators

**Outcome Rate the importance of the following :**	**Category**	**Overall Significance** [a]	**Post hoc analysis**					
			**High-Marginal vs. Very Low Food Security**		**High-Marginal vs. Low Food Security**		**Low Food Security vs. Very Low Food Security**	
			**OR (95% CI)**	***p*-value**	**OR (95% CI)**	***p*-value**	**OR (95% CI)**	***p*-value**
Low calorie (kilojoule)		0.745						
	Important		0.91 (0.53, 1.56)	0.740	0.84 (0.52, 1.37)	0.493	1.08 (0.578, 2.02)	0.806
	Neither important nor unimportant		0.80 (0.45, 1.42)	0.450	0.99 (0.58, 1.70)	0.980	0.81 (0.41, 1.59)	0.534
	Unimportant		1.00 (ref)		1.00 (ref)		1.00 (ref)	
High protein		0.092						
	Important		0.50 (0.26, 0.93)	0.029**	0.58 (0.34, 0.99)	0.047*	0.87 (0.42, 1.80)	0.706
	Neither important nor unimportant		0.57 (0.30, 1.09)	0.090	0.72 (0.40, 1.27)	0.251	0.8 (0.37, 1.72)	0.567
	Unimportant		1.00 (ref)		1.00 (ref)		1.00 (ref)	
Low saturated fats		0.860						
	Important		0.74 (0.39, 1.41)	0.352	0.85 (0.48, 1.52)	0.582	0.87 (0.41, 1.84)	0.707
	Neither important nor unimportant		0.69 (0.34, 1.41)	0.303	0.80 (0.42, 1.53)	0.501	0.86 (0.37, 1.98)	0.716
	Unimportant		1.00 (ref)		1.00 (ref)		1.00 (ref)	
Low carbohydrates		0.055						
	Important		0.56 (0.32, 0.99)	0.046	0.82 (0.50, 1.32)	0.409	0.69 (0.36, 1.32)	0.256
	Neither important nor unimportant		0.87 (0.48, 1.57)	0.638	1.17 (0.71, 1.95)	0.527	0.73 (0.37, 1.46)	0.381
	Unimportant		1.00 (ref)		1.00 (ref)		1.00 (ref)	
Low sodium		0.951						
	Important		1.09 (0.55, 1.85)	0.981	1.10 (0.64, 1.89)	0.738	0.92 (0.46, 1.84)	0.810
	Neither important nor unimportant		0.98 (0.51, 1.89)	0.945	1.24 (0.68, 2.26)	0.492	0.79 (0.37, 1.69)	0.544
	Unimportant		1.00 (ref)		1.00 (ref)		1.00 (ref)	
Low sugar		0.672						
	Important		1.23 (0.65, 2.33)	0.521	1.3 (0.72, 2.33)	0.380	0.95 (0.46, 1.95)	0.884
	Neither important nor unimportant		1.45 (0.71, 2.97)	0.313	1.58 (0.81, 3.08)	0.178	0.92 (0.40, 2.08)	0.834
	Unimportant		1.00 (ref)		1.00 (ref)		1.00 (ref)	

**Outcome**<br>**Rate the importance of the following :**	**Category**	**Overall Significance** [a]	**Post hoc analysis**					
			**High-Marginal vs. Low Food Security**		**High-Marginal vs. Very Low Food Security**		**Low Food Security vs. Very Low Food Security**	
			**OR (95% CI)**	***p*-value**	**OR (95% CI))**	***p*-value**	**OR (95% CI))**	***p*-value**
Low glycemic index		0.261						
	Important		0.71 (0.41, 1.23)	0.222	0.58 (0.34, 0.99)	0.047	1.23 (0.62, 2.43)	0.556
	Neither important nor unimportant		0.87 (0.49, 1.55)	0.629	0.67 (0.38, 1.17)	0.157	1.30 (0.64, 2.64)	0.466
	Unimportant		1.00 (ref)		1.00 (ref)		1.00 (ref)	
Low preservatives		0.120						
	Important		0.54 (0.26, 1.10)	0.090	0.81 (0.33, 2.01)	0.652	1.231 (0.50, 3.04)	0.652
	Neither important nor unimportant		0.61 (0.29, 1.31)	0.206	0.47 (0.22, 1.01)	0.052	1.30 (0.50, 3.35)	0.590
	Unimportant		1.00 (ref)		1.00 (ref)		1.00 (ref)	

[a] Multinomial logistic regression model was adjusted for socio-demographics variables (age, household income, education and marital status)

* p-value < 0.05; ** p-value < 0.01; OR = odds ratio; CI = confidence interval; 1.00 (ref) = reference level

Table S6: Response, by three categories of food security p-values and odds ratios of product attribute indicators

**Outcome**	**Category**	**Overall Significance** [a]	**Post hoc analysis**					
			**High-Marginal vs. Very Low Food Security**		**High-Marginal vs. Low Food Security**		**Low Food Security vs. Very Low Food Security**	
			**OR (95% CI))**	***p*-value**	**OR (95% CI)**	***p*-value**	**OR (95% CI)**	***p*-value**
Nutrition		0.021*						
	Important		1.00 (ref)		1.00 (ref)		1.00 (ref)	
	Neither important nor unimportant		0.49 (0.30, 0.81)	0.005**	0.53 (0.34, 0.84)	0.006**	0.93 (0.54, 1.59)	0.790
	Unimportant		0.73 (0.28, 1.91)	0.531	0.82 (0.33, 2.05)	0.667	0.90 (0.30,2.69)	0.853
Quality		0.010*						
	Important		1.00 (ref)		1.00 (ref)		1.00 (ref)	
	Neither important nor unimportant		0.40 (0.20, 0.81)	0.011*	0.40 (0.21, 0.76)	0.005**	1.00 (0.47, 2.11)	0.994
	Unimportant		0.36 (0.10, 1.28)	0.115	0.383 (0.12, 1.28)	0.119	0.94 (0.25, 3.53)	0.932
Cost		0.117						
	Important		1.00 (ref)		1.00 (ref)		1.00 (ref)	1.00 (ref)
	Neither important nor unimportant		2.19 (1.08, 4.46)	0.031*	1.41 (0.80, 2.49)	0.234	1.55 (0.68, 3.55)	0.298
	Unimportant		0.61 (0.23, 1.64)	0.327	0.74 (0.30, 1.82)	0.507	0.83 (0.27, 2.60)	0.749
In season		0.614						
	Important		1.14 (0.55, 2.38)	0.720	1.47 (0.78, 2.77)	0.229	0.78 (0.341, 1.77)	0.545
	Neither important nor unimportant		1.44 (0.65, 3.23)	0.372	1.44 (0.72, 2.88)	0.304	1.00 (0.41, 2.48)	0.994
	Unimportant		1.00 (ref)		1.00 (ref)		1.00 (ref)	
Local product		0.205						
	Important		1.81 (0.98, 3.37)	0.060	1.444 (0.784, 2.66)	0.238	1.26 (0.62, 2.54)	0.528
	Neither important nor unimportant		2.28 (1.15, 4.52)	0.018*	1.42 (0.73, 2.73)	0.300	1.61 (0.74, 3.51)	0.229
	Unimportant		1.00 (ref)		1.00 (ref)		1.00 (ref)	
Organic		0.027*						
	Important		0.65 (0.42, 1.01)	0.056	0.55 (0.36, 0.83)	0.005**	1.19 (0.70, 2.00)	0.520
	Neither important nor unimportant		1.01 (0.66, 1.56)	0.963	0.73 (0.49, 1.08)	0.116	1.39 (0.83, 2.33)	0.205
	Unimportant		1.00 (ref)		1.00 (ref)		1.00 (ref)	

Outcome	Category	Overall Significance [a]	Post hoc analysis					
			High-Marginal vs. Very Low Food Security		High-Marginal vs. Low Food Security		Low Food Security vs. Very Low Food Security	
			OR (95% CI)))	*p*-value	OR (95% CI))	*p*-value	OR (95% CI))	*p*-value
Raw food (natural state)		0.024*						
	Important		1.119 (0.71, 1.77)	0.632	0.57 (0.35, 0.93)	0.023	1.96 (1.10, 3.47)	0.022*
	Neither important nor unimportant		1.60 (0.98, 2.62)	0.059	0.77 (0.47, 1.273)	0.309	2.08 (1.13, 3.82)	0.018*
	Unimportant		1.00 (ref)		1.00 (ref)		1.00 (ref)	
Unprocessed		0.521						
	Important		1.30 (0.77, 2.18)	0.329	0.91 (0.54, 1.55)	0.735	1.42 (0.76, 2.65)	0.276
	Neither important nor unimportant		1.59 (0.91, 2.78)	0.101	0.95 (0.55, 1.65)	0.862	1.67 (0.86, 3.26)	0.131
	Unimportant		1.00 (ref)		1.00 (ref)		1.00 (ref)	
Convenience (pre-packaged to save time) e.g. pre-cut vegetables, pre-marinated meats, bottle sauces		<0.001**						
	Important		0.53 (0.34, 0.83)	0.005**	0.40 (0.27, 0.61)	<0.001**	1.31 (0.77, 2.22)	0.325
	Neither important nor unimportant		0.55 (0.36, 0.85)	0.007	0.52 (0.35, 0.79)	0.002**	1.06 (0.63, 1.78)	0.833
	Unimportant		1.00 (ref)		1.00 (ref)		1.00 (ref)	
Australian grown		0.889						
	Important		1.36 (0.71, 2.60)	0.359	1.09 (0.58, 2.04)	0.794	1.25 (0.59, 2.65)	0.564
	Neither important nor unimportant		1.23 (0.59, 2.56)	0.582	1.18 (0.58, 2.40)	0.658	1.046 (0.45, 2.46)	0.918
	Unimportant		1.00 (ref)		1.00 (ref)		1.00 (ref)	
Supermarket branded (homebrand, Coles Select)		<0.001**						
	Important		0.37 (0.23, 0.60)	<0.001**	0.214 (0.131, 0.349)	<0.001**	1.74 (0.97, 3.12)	0.066
	Neither important nor unimportant		0.83 (0.53, 1.28)	0.391	0.475 (0.303, 0.743)	0.001**	1.74 (0.99, 3.05)	0.053
	Unimportant		1.00 (ref)		1.00 (ref)		1.00 (ref)	

[a] Multinomial logistic regression model was adjusted for socio-demographics variables (age, household income, education and marital status)

* p-value < 0.05; ** p-value < 0.01; OR = odds ratio; CI = confidence interval; 1.00 (ref) = reference level

Table S7: Response, by three categories of food security, frequencies and proportions for consumption behaviours

Question		Serves	High-Marginal Food Security	Low Food Security	Very Low Food Security
On a typical day, how many serves [a] of the following foods would you eat?					
Breakfast cereals	2/3 cup breakfast cereals, cooked oats	0	119 (18%)	40 (20%)	41 (24%)
	2 weet-biscuits	1	476 (73%)	143 (70%)	117 (68%)
		2 or more	56 (9%)	21 (10%)	13 (8%)
Milk, yoghurt, cheese and dairy alternatives	1 cup of milk or soy milk	0	41 (6%)	18 (9%)	17 (10%)
	2 slices of cheese	1	358 (55%)	115 (56%)	95 (56%)
	1 tub of yoghurt	2	194 (30%)	49 (24%)	45 (26%)
		3 or more	58 (9%)	22 (11%)	14 (8%)
Bread	1 slice of bread	0	39 (6%)	12 (6%)	20 (12%)
	1 crumpet or English muffin	1	248 (38%)	89 (44%)	77 (45%)
		2	294 (45%)	76 (37%)	58 (34%)
		3 or more	70 (11%)	27 (13%)	16 (9%)
Fruit (not including juice)	1 medium banana, apple or orange	0	27 (4%)	11 (5%)	18 (11%)
	2 small kiwi fruit, apricots or plums	1	297 (46%)	102 (50%)	81 (47%)
	1 cup canned fruit	2	217 (33%)	63 (31%)	47 (27%)
	A handful of dried fruit (e.g. 4 apricot halves)	3 or more	110 (17%)	28 (14%)	25 (15%)
Fruit (juice)	1 cup fruit juice	0	186 (29%)	48 (24%)	54 (32%)
		1	392 (60%)	120 (59%)	86 (50%)
		2 or more	73 (11%)	36 (18%)	31 (18%)

Question		Serves	High-Marginal Food Security	Low Food Security	Very Low Food Security
On a typical day, how many serves [a] of the following foods would you eat?					
Salad and vegetables (not including potato)	1 cup salad vegetables (e.g. lettuce, cucumber, tomato)	0	15 (2%)	14 (7%)	15 (9%)
		1	289 (44%)	100 (49%)	74 (43%)
	½ cup cooked or canned vegetables	2	198 (30%)	49 (24%)	45 (26%)
		3	92 (14%)	31 (15%)	25 (15%)
		4 or more	57 (9%)	10 (5%)	12 (7%)
Potato (not including chips)	½ medium potato	0	35 (5%)	21 (10%)	24 (14%)
	½ cup mashed potato	1	506 (78%)	142 (70%)	115 (67%)
		2 or more	110 (17%)	41 (20%)	32 (19%)
Pasta, rice, or noodles	½ cup cooked pasta or rice, noodles	0	34 (5%)	21 (10%)	19 (11%)
		1	487 (75%)	136 (67%)	116 (68%)
		2 or more	130 (20%)	47 (23%)	36 (21%)
Meat alternatives	1 cup baked beans, cooked legumes or tofu	0	64 (10%)	30 (15%)	27 (16%)
	2 large eggs	1	499 (77%)	147 (72%)	118 (69%)
		2 or more	88 (14%)	27 (13%)	26 (15%)
Fish	A cooked fish fillet about the size of an open hand (100g)	0	81 (12%)	26 (13%)	34 (20%)
		1	488 (75%)	144 (71%)	115 (67%)
	One small can of fish (100g)	2 or more	82 (13%)	34 (17%)	22 (13%)
Poultry	Cooked lean poultry such as chicken or turkey, about the size of an open hand (80g)	0	38 (6%)	11 (5%)	20 (12%)
		1	497 (76%)	141 (69%)	120 (70%)
		2 or more	116 (18%)	52 (25%)	31 (18%)

Question		Serves	High-Marginal Food Security	Low Food Security	Very Low Food Security
On a typical day, how many serves [a] of the following foods would you eat?					
Red meat	Cooked lean meat, about the size of a deck of playing cards (65g)	0	57 (9%)	18 (9%)	20 (12%)
		1	472 (73%)	142 (70%)	122 (71%)
		2 or more	122 (19%)	44 (22%)	29 (17%)
Nuts and seeds	A handful of nuts /seeds	0	93 (14%)	35 (17%)	46 (27%)
		1	450 (69%)	123 (60%)	89 (52%)
		2 or more	108 (17%)	46 (23%)	36 (21%)
Savoury snacks	2 slices of processed meat	0	96 (15%)	28 (14%)	33 (19%)
	12 hot chips	1	440 (68%)	134 (66%)	102 (60%)
	½ small packet of crisps (20g)	2 or more	115 (18%)	42 (21%)	36 (21%)
Sweet snacks	2 scoops ice cream	0	55 (8%)	25 (12%)	27 (16%)
	1 doughnut, slice of cake, muffin	1	474 (73%)	137 (67%)	107 (63%)
	½ regular bar of chocolate (25g)	2 or more	122 (19%)	42 (21%)	37 (22%)
	2-3 biscuits				
Water (including tea and coffee)	1 cup (250ml)	0	11 (2%)	12 (6%)	15 (9%)
		1	103 (16%)	47 (23%)	35 (20%)
		2	73 (11%)	24 (12%)	21 (12%)
		3	88 (14%)	24 (12%)	28 (16%)
		4 or more	376 (58%)	97 (48%)	72 (42%)

Question		Serves	High-Marginal Food Security	Low Food Security	Very Low Food Security
On a typical day, how many serves [a] of the following foods would you eat?					
Additional drinks (not including alcohol)	1 can of soft drink (375ml)	0	170 (26%)	42 (21%)	43 (25%)
	2 cups of cordial (500ml)	1	371 (57%)	111 (54%)	82 (48%)
	1 can energy drink (330 ml)	2	81 (12%)	34 (17%)	28 (16%)
	2 cups of Sports drink (500 ml)	3 or more	29 (4%)	17 (8%)	18 (11%)
Alcohol	30 ml spirits	0	156 (24%)	57 (28%)	57 (33%)
	60 ml fortified wine	1	233 (36%)	73 (36%)	61 (36%)
	100 ml wine	2	142 (22%)	38 (19%)	34 (20%)
	425 ml light beer	3	53 (8%)	16 (8%)	12 (7%)
	285 ml regular beer	4 or more	67 (10%)	20 (10%)	7 (4%)
	Small bottle of premix drink or 'alco-pop' (300 ml)				

[a] Serves as defined by the Australian Dietary Guidelines
